# Thrombomodulin: A Bifunctional Modulator of Inflammation and Coagulation in Sepsis

**DOI:** 10.1155/2012/614545

**Published:** 2012-02-28

**Authors:** Takayuki Okamoto, Hironobu Tanigami, Koji Suzuki, Motomu Shimaoka

**Affiliations:** ^1^Department of Molecular Pathobiology and Cell Adhesion Biology, Graduate School of Medicine, Mie University, 2-174 Edobashi, Tsu 514-8507, Japan; ^2^Department of Anesthesiology, Osaka Medical Center for Cancer and Cardiovascular Diseases, 1-3-3 Nakamichi, Higashinari-ku, Osaka 537-8511, Japan; ^3^Faculty of Pharmaceutical Science, Suzuka University of Medical Science, 3500-3 Minamitamagaki-cho, Suzuka City 513-8679, Japan

## Abstract

Deregulated interplay between inflammation and coagulation plays a pivotal role in the pathogenesis of sepsis. Therapeutic approaches that simultaneously target both inflammation and coagulation hold great promise for the treatment of sepsis. Thrombomodulin is an endogenous anticoagulant protein that, in cooperation with protein C and thrombin-activatable fibrinolysis inhibitor, serves to maintain the endothelial microenvironment in an anti-inflammatory and anticoagulant state. A recombinant soluble form of thrombomodulin has been approved to treat patients suffering from disseminated intravascular coagulation (DIC) and has thus far shown greater therapeutic potential than heparin. A phase II clinical trial is currently underway in the USA to study the efficacy of thrombomodulin for the treatment of sepsis with DIC complications. This paper focuses on the critical roles that thrombomodulin plays at the intersection of inflammation and coagulation and proposes the possible existence of interactions with integrins via protein C. Finally, we provide a rationale for the clinical application of thrombomodulin for alleviating sepsis.

## 1. Introduction

Septic shock is a leading cause of death in intensive care units and thus represents a substantial financial burden on the healthcare system in Japan, in the United States, and in many other developed nations [1, 2]. Although the mortality rate of septic shock has tended to gradually decrease during the past decade thanks to significant technological advances in supportive therapies, it remains high and effective specific treatments are still very limited [3]. Uncontrolled and systemically spreading inflammatory responses that are initiated by infection play critical roles in the pathogenesis of septic shock. At sites of uncontrolled inflammation, endothelial cells are damaged, thereby upregulating intercellular-adhesion-molecule-1 (ICAM-1) and vascular-cell-adhesion-molecule-1 (VCAM-1) expression, which not only enables the accumulation of leukocytes but also heightens permeability, thus leading to tissue edema formation. Damaged endothelial cells exhibit morphological abnormalities such as nuclear vacuolation, protrusion, and cytoplasmic fragmentation, thereby being subjected to the detachment from the basement membrane [4]. Furthermore, inflammation deregulates coagulation cascades, thereby inducing intravascular blood coagulation at inflamed endothelial cells, which reflects the tendency of septic shock to manifest various coagulopathies that lead to disseminated intravascular coagulation (DIC) [5].

Current consensus on septic shock pathogenesis holds that dysregulation occurs simultaneously in both the inflammation and coagulation systems, thereby complicating pathogenesis in patients and reducing the therapeutic effectiveness of targeting either inflammation or coagulation. A better understanding of the crosstalk that occurs between inflammation and coagulation is therefore critical to developing novel, effective treatments for sepsis. Recent investigations have shown that activated protein C (APC), an endogenous anticoagulant protein, possesses both anti-inflammatory and anticoagulant properties. A recombinant form of APC (Drotrecogin alfa-activated (DrotAA)) has been used in the treatment of severe septic shock [6]. In this paper, we focus on another important endogenous anticoagulant protein, thrombomodulin (TM). Recombinant soluble TM (Recomodulin) has been approved in Japan in 2008 for the treatment of DIC resulting from infection and cancer [7–11]. Currently in the USA a phase II clinical trial involving sepsis DIC patients has been completed and subsequent phase III trials are planned. Here, we examine the basic biology of TM with a particular emphasis on its role at the intersection of inflammation and coagulation and then discuss its potential application for septic shock.

## 2. Crosstalk between Inflammation and Coagulation

**Figure 1:**
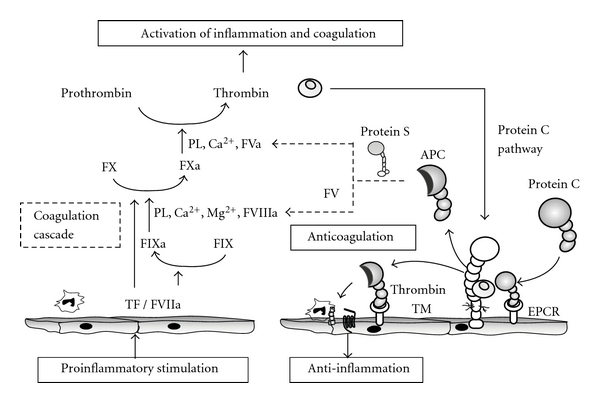
Crosstalk between inflammation and coagulation. Proinflammatory stimulation induces TF expression in monocytes, macrophages, and endothelial cells. TF initiates a coagulation cascade and the FXa-FVa complex converts thrombin. Thrombin induces fibrin formation and activates platelets, monocytes, and endothelial cells through PARs. Thus, thrombin acts to form a positive feedback loop that augments coagulation and inflammation. In a negative feedback loop, the thrombin-TM complex activates protein C and downregulates coagulation and inflammation. APC, in tandem with its cofactor protein S, inactivates FVa and FVIIIa. In addition, the APC-EPCR complex induces anti-inflammatory effects through PARs. Therefore, thrombin, TM, and protein C-EPCR play pivotal roles in the crosstalk that occurs between inflammation and coagulation.

Systemic inflammation causes the activation of the coagulation cascades, and, vice versa, the players in the coagulation cascades also possess the abilities to modulate inflammation [12]. The interplays between inflammation and coagulation are at the center of the pathogenesis underlying septic shock (Figure 1) [5, 13]. One of the major pathways in which inflammation augments blood coagulation is the generation of thrombin mediated by tissue factor (TF) that is upregulated on monocytes, macrophages, and endothelial cells [14]. Endotoxin and proinflammatory cytokines (e.g., interleukin-6 (IL-6)) induce the expression of TF, which binds to factor VII (FVII), thus forming the TF/FVIIa complex that activates FX. FXa assembles with FVa, thereby generating a prothrombinase complex on the surface of endothelial cells and monocytes. This prothrombinase complex converts prothrombin to thrombin, which in turn converts fibrinogen to fibrin, and thereby leads to fibrin formation and platelet activation and aggregation. In addition, inflammation impairs functions of important anticoagulant pathways that are governed by antithrombin, protein C, and tissue factor pathway inhibitor (TFPI). Conversely, inflammation is modified by important players in coagulation and anti-coagulation pathways such as thrombin, antithrombin, thrombomodulin, and protein C as well as fibrinogen and fibrin modulate inflammation [12]. For example, thrombin and other activated coagulation factors exhibit an array of proinflammatory activities via cleavage activation of protease-activated receptors (PARs) [15, 16]. Thrombin induces the following: P-selectin expression in endothelial cells, monocyte and neutrophil chemotaxis [17, 18], leukocyte adhesion molecule expression [15], IL-6 and IL-8 production by endothelial cells [19], and lymphocyte and monocyte activation and proliferation [20, 21].

**Figure 2:**
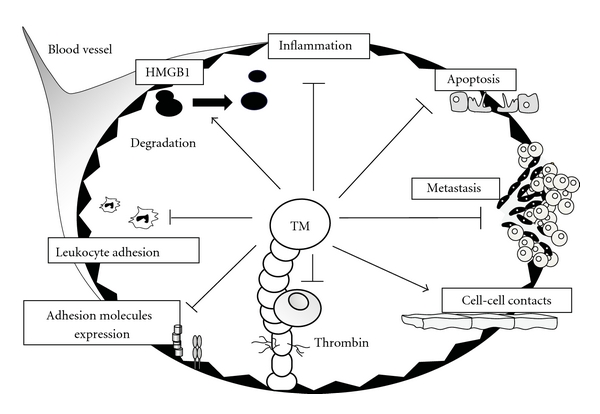
Multiple roles of TM in the maintenance of vascular homeostasis. TM inhibits inflammation, apoptosis, tumor metastasis, thrombin function, adhesion molecule expression, and leukocyte adhesion. Proinflammatory high-mobility group box 1 (HMGB1) is neutralized and degraded by TM.

While thrombin formation is the driving force behind procoagulation and proinflammatory states, thrombin itself remains tightly regulated and under the control of negative feedback loops in which TM plays a critical role in tandem with protein C (Figure 1) [5, 22]. The interaction of TM with thrombin switches thrombin substrate specificity from fibrinogen to protein C [23]. Thrombin, TM, and protein C bind simultaneously, thereby forming a complex on the surface of the endothelium that expresses endothelial cell protein C receptor (EPCR). During the course of complex formation, protein C is converted to its active form: activated PC (APC) [24]. APC subsequently dissociates from the APC-EPCR complex and binds to protein S, in this way forming an APC-protein S complex. The APC-protein S complex dampens not only coagulation, by inactivating FVa and FVIIIa, but also inflammation, by inhibiting the production of inflammatory cytokines and the transduction of nuclear-factor- (NF-)*κ*B signaling in monocytes [25]. Furthermore, APC suppresses LPS-induced increases in the pulmonary accumulation of leukocytes and in vascular permeability [26]. Therefore, the protein C pathway plays a pivotal role in the negative regulation of coagulation and inflammation. The thrombin-TM complex also activates the thrombin-activatable fibrinolysis inhibitor (TAFI), which in turn inhibits fibrinolysis and inactivates anaphylatoxin C3a and C5a [27, 28]. In addition to acting as a cofactor for APC and TAFI activation, TM has exhibited a wide range of biological activities in maintaining vascular homeostasis (Figure 2) [29].

## 3. Structure and Domains of Thrombomodulin

**Figure 3:**
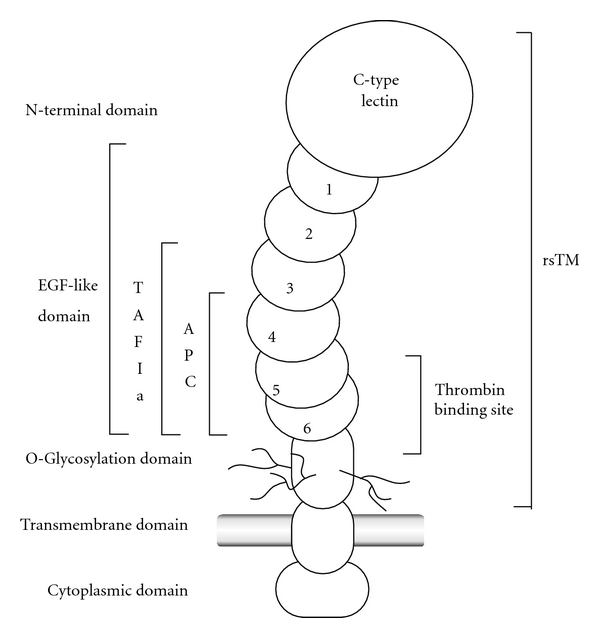
Structure and domains of TM. TM contains an N-terminal C-type lectin-like domain, an extended stalk of six EGF modules, a serine/threonine-rich region with target sites for posttranslational glycosylation, a transmembrane domain, and a short cytoplasmic domain. EGF domains 5-6 bind thrombin. EGF4-6 is required for protein C activation and EGF3-6 for TAFI activation.

Human TM is a single-chain type 1 transmembrane glycoprotein measuring 557-amino acid residues long and containing five extracellular domains (Figure 3) [30, 31]. The N-terminal region of TM comprises about half of the extracellular portion of the molecule and contains a module with a homology to other C-type lectins. Although this domain lacks anticoagulant activity, it plays an important role in mediating anti-inflammatory activities [32, 33].

The next domain of TM contains six epidermal-growth-factor-(EGF-) like repeats that form an extended stalk in the extracellular part of molecule. This domain showed mitogenic activities on cultured fibroblasts and vascular smooth muscle cells. The activities were mediated via activation of protein kinase C and mitogen-activated protein kinase (MAPK) [34, 35]. The interaction of the EGF5-6 repeats with thrombin has been shown to prevent the binding of procoagulant substrates (e.g., such as FV, fibrinogen) to thrombin [36–39]. These EGF4-6 repeats are required for the activation of protein C, while the EGF3-6 repeats are needed for the activation of TAFI [39–41].

The third domain is a serine/threonine-rich domain bearing potential sites for *O*-linked glycosylation. This domain supports the attachment of chondroitin sulfate. Biochemical studies have shown that chondroitin sulfate, when attached to TM, enhanced the potency of the latter to activate protein C [42, 43]. In addition, it accelerates the neutralization of thrombin and facilitates the binding of platelet factor 4 (PF4) to protein C [44]. The chondroitin sulfate moiety may strengthen the thrombin-TM binding by interacting with the anion binding exosite of thrombin, to which heparin binds. Of note, heparin was shown to inhibit the thrombin-TM binding possibly by competing with the TM chondroitin sulfate moiety for the thrombin exosite [45].

A well-conserved transmembrane domain is typically followed by a short cytoplasmic tail. A cysteine in the cytoplasmic tail is thought to mediate the multimerization of TM [46]. In human-cultured vein endothelial cells, thrombin binding to TM induced signaling events that lead to the activation of endothelial nitric oxide (NO) synthase 3 (NOS-3) through modulation of G protein-coupled receptor (GPCR) signals [47]. Although knockout mice completely lacking TM died *in utero* due to a defect in placental development [48], knock-in mice expressing mutant TM that lacks the cytoplasmic domain showed no abnormalities in fetal development, survival, and coagulation [49, 50].

Recomodulin, a recombinant form of human soluble TM, comprises only the extracellular domain of TM that includes N-terminal C-type lectin domain, EGF-like domain, and *O*-glycosylation domain [51]. Similar to a native membrane-bound TM, rhsTM binds to thrombin to inactivate coagulation, and thrombin-rhsTM complex activates protein C to produce APC.

## 4. Anticoagulant Properties of Thrombomodulin

TM exerts its anticoagulant activity not only by inhibiting thrombin but also by accelerating APC generation [36, 37, 52, 53]. Upon TM binding, thrombin undergoes a reduction in its affinity to procoagulant substrates [36, 37, 52, 53]. TM directly inhibits most of the procoagulant functions of thrombin including fibrinogen clotting, platelet and EC activation, and FV activation [36, 37]. In addition, TM accelerates the inactivation of thrombin via both antithrombin and protein C inhibitor [54, 55]. TM switches thrombin substrates specificity to protein C [23]. APC suppresses further thrombin formation by proteolytically degrading FVa and FVIIIa. This activity is enhanced by protein S, the cofactor for APC (Figure 1). The anti-inflammatory properties of TM may be partly explained by the concept that the affinity of thrombin for TM is likely much higher than that for other factors in pro- and anticoagulant pathways [23], potentially making TM a scavenging inhibitor of circulating thrombin.

Inflammation has been reported to downregulate TM expression [56]. Tumor necrosis factor-*α*  (TNF-*α*) induces internalization of TM via endocytosis, thereby reducing its surface expression [57]. Such reduced TM expression at sites of inflammatory injury may exacerbate blood coagulation. Indeed, endothelium-specific deletion of TM in mice caused spontaneous and fatal thrombosis in the arterial and venous vessels [58], indicating that TM may play a role in preventing intravascular thrombus formation.

## 5. Anti-Inflammatory Effects of Thrombomodulin

### 5.1. APC-Dependent Mechanisms

**Figure 4:**
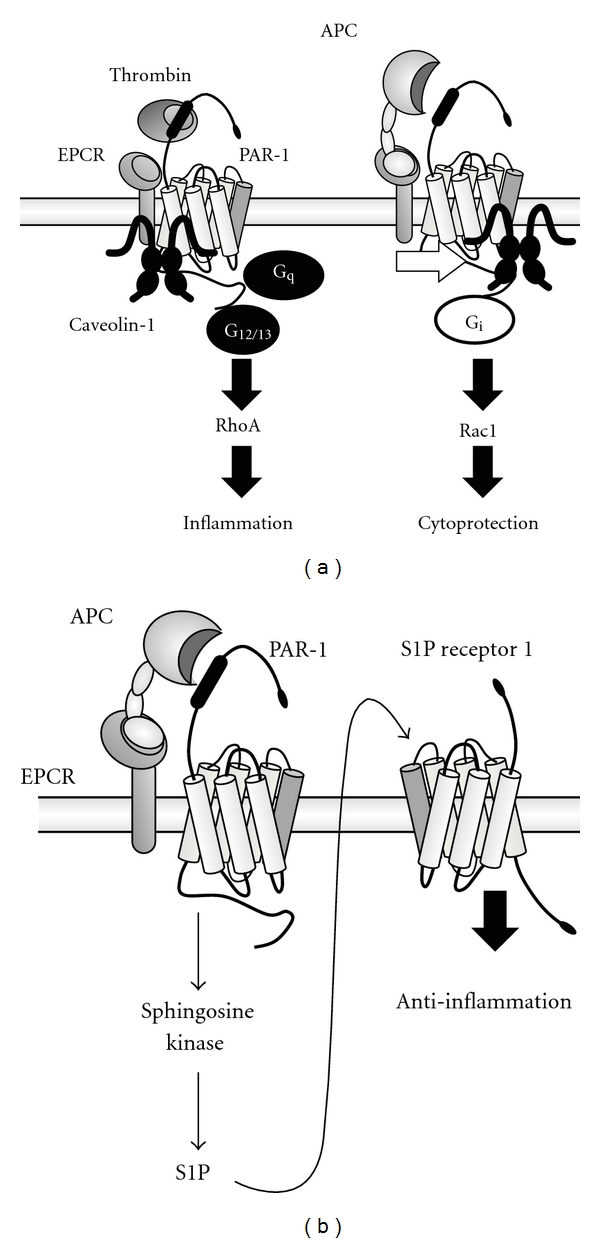
Potential molecular mechanisms by which APC executes anti-inflammatory effects. (a) Caveolin-1 is associated with EPCR in lipid rafts in the absence of protein C or APC. Thrombin cleavage of PAR-1 induces inflammatory responses by coupling receptors to G12/13 and Gq proteins. The occupancy of EPCR by protein C results in the dissociation of EPCR from caveolin-1. Thrombin induces a cytoprotective response by coupling receptors to Gi protein. (b) The APC-EPCR complex cleavage of PAR-1 induces S1P generation and S1P receptor 1 transactivation. Activation of S1P receptor 1 improves endothelial barrier function, thus facilitating the ability of the APC-EPCR complex to exert anti-inflammatory effects.

TM has been shown to mediate anti-inflammatory activities using APC-dependent and APC-independent mechanisms, depending on the setting [22]. In the APC-dependent mechanism, TM executes its anti-inflammatory activities by enhancing the activation of protein C. The resulting APC downregulates inflammatory cytokine production by decreasing the expression of NF-*κ*B components and inhibiting their nuclear translocation [59–62]. These anti-inflammatory effects are dependent upon APC, EPCR, and PAR-1 [63, 64]. It is thought that protein C and/or APC binding to EPCR results in the coupling of PAR-1 to Gi in endothelial cells (Figure 4(a)) [65–67]. Both PAR-1 and EPCR are known to associate with caveolin-1 in lipid rafts or protein C- or APC-EPCR complexes, leading to caveolin-1 dissociation from EPCR. This process appears to favor anti-inflammatory phenotypes of endothelial cells [68].

APC has been shown to enhance the endothelial barrier function through transactivation of sphingosine-1-phosphate (S1P) receptor signaling (Figure 4(b)) [69, 70]. Improving endothelial barrier function would provide anti-inflammatory effects by reducing leukocyte extravasation to sites of inflammation. Apolipoprotein E receptor 2 (ApoER2) and angiopoietin (Ang)/tie2 signaling pathways have been shown to contribute to the cytoprotective and anti-inflammatory properties of APC [71, 72].

In addition to acting on endothelial cells, APC has been shown to target leukocytes. In one study, APC bound to leukocyte integrins through an RGD motif, thereby inhibiting neutrophil migration into inflamed tissues [73]. In addition, APC was shown to cleave and consequently neutralize the cytotoxicity of extracellular histones released from dying leukocytes [74]. Histone administration resulted in neutrophil margination, microhemorrhage, intravascular thrombosis, and capillary leak of fibrin. Coadministration of APC alleviated lethality of histone. By contrast, histone toxicity was much more pronounced in mice, where the PC pathway was blocked [74].

Reduced levels of PC were found in the majority of patients with sepsis and were associated with higher rates of morbidity and mortality [75, 76]. A controversial PROWESS study showed that treatment with recombinant APC not only lowered the plasma levels of D-dimer and IL-6 but also reduced mortality in patients with severe sepsis [6].

### 5.2. APC-Independent Mechanisms

**Figure 5:**
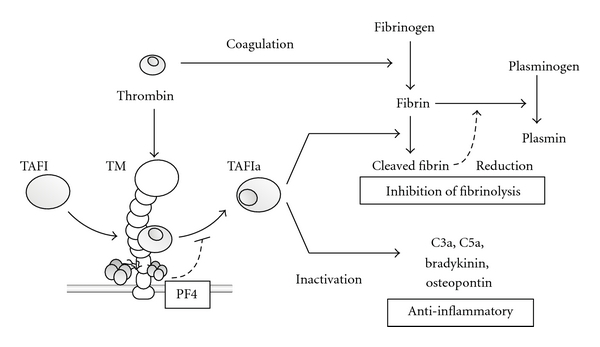
TAFIa-dependent modulation of inflammation and fibrinolysis. TAFIa appears to possess anti-inflammatory properties due to its ability to inactivate proinflammatory mediators, C3a, C5a, bradykinin, and osteopontin by removing their C-terminal residues. TAFIa also removes the C-terminal lysines of fibrin, leading to a reduction in the rates of plasmin generation and fibrinolysis. PF4 inhibits the activation of TAFI and enhances the activation of protein C by the TM-thrombin complex.

TM modulates fibrinolysis and inflammation through thrombin-activatable fibrinolysis inhibitor (TAFI). TAFI is a circulating zymogen that is activated by the thrombin-TM complex [77, 78]. Activated TAFI eliminates lysine residues at the C-terminal end of fibrin, thereby suppressing the incorporation of plasminogen and tissue plasminogen activator (t-PA) into the fibrin clot (Figure 5). Thus, activation of TAFI by TM inhibits fibrinolysis, thereby preventing the resolution of already formed clots. This activity complements the role of TM-induced activated protein C to prevent the formation of new clots, thereby balancing hemostasis [79]. While diminishing fibrinolysis by acting on fibrin, activated TAFI also affects inflammation [77, 78]. TAFIa has been reported to act on, and inactivate, proinflammatory mediators such as the anaphylatoxins complement factors C3a and C5a [80–82], bradykinin, and osteopontin [44, 78].

**Figure 6:**
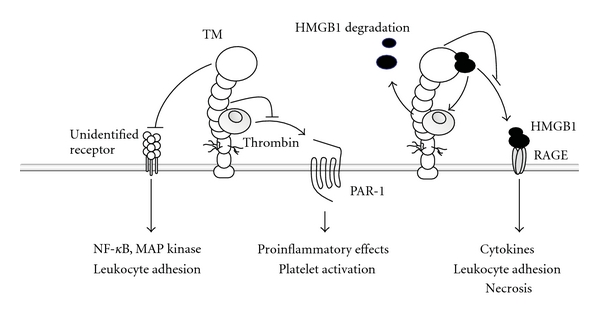
APC- and TAFIa-independent anti-inflammatory effects of TM. Thrombomodulin binds to thrombin, thereby blocking activation of protease-activated receptors. The lectin-like domain binds to, and enhances degradation of, proinflammatory molecule HMGB1. In addition, the lectin-like domain is thought to bind to an unidentified receptor, thereby suppressing MAP kinase and NF-*κ*B activation.

Independently of APC, TM exhibits anti-inflammatory activities via the EGF domain of TM and the N-terminal lectin-like domain (Figure 6) [22, 83]. TM binds to thrombin, thereby inhibiting the proinflammatory activities of thrombin [16], including induction of endothelial-inducible nitric oxide synthase expression, monocyte and neutrophil chemotactic activity, upregulation of leukocyte adhesion molecules, enhancement of IL-6 and IL-8 production from endothelial cells, and mitogenic activity involving lymphocytes, fibroblasts, macrophages, mesangial cells, neuroblastoma cells, and osteoblasts. Several of thrombin proinflammatory properties are mediated via the cleavage and activation of PARs [16]. TM binding to thrombin has been shown to decrease PAR-1-induced activation of ERK1/2 and to suppress the mitogenic effects of thrombin [33, 84].

The anti-inflammatory activities of the N-terminal lectin-like domain are thought to act in an APC-independent manner, since this domain is dispensable to APC generation. Mutant mice expressing TM that lacks this lectin-like domain did not show altered *in vivo* APC generation [33]. However, these mice did exhibit an increased susceptibility to endotoxin-induced sepsis. Endothelial cells isolated from mutant mice exhibited elevated ICAM-1 expression as well as increased ICAM-1-mediated leukocyte adhesion. In good agreement with these findings, a soluble lectin-like N-terminal domain of TM proved sufficiently robust to inhibit TNF-*α*-induced ERK phosphorylation and restore normal leukocyte adhesion to mutant endothelial cells. In addition, this recombinant lectin-like TM domain also protected cultured endothelial cells from cell death, possibly via modulation of NF-*κ*B pathways, but more likely by serum deprivation [33].

Another anti-inflammatory target of TM is high-mobility group box 1 (HMGB1), a ubiquitously expressed nuclear protein that is released from necrotic cells. Upon being released, HMGB1 binds to the receptor for advanced glycation end products (RAGE) [85]. HMGB1-RAGE signaling has been implicated in the pathogenesis and/or progression of various clinical disorders, such as infections, sepsis, arthritis, and cancer. The lectin-like domain of TM interferes with HMGB1 binding to RAGE, thereby impairing HMGB1-RAGE signaling [86]. Alternatively, TM may antagonize HMGB1 by enhancing thrombin-mediated proteolytic degradation of HMGB1 [87].

## 6. Therapeutic Application of TM

The dual ability of TM to suppress both coagulation and inflammation makes this molecule a promising drug candidate for the treatment of DIC and/or septic shock. In order to administer it intravenously, a soluble form of recombinant TM (rhsTM), containing only the extracellular domains, has been developed [88]. Like the membrane-bound native TM, rhsTM retains the ability to bind to thrombin and APC. Administration of rhsTM has been shown to protect rats from TF and endotoxin-induced DIC or lung injury [89–91]. In addition, rhsTM not only reduced compression trauma-induced spinal cord injury by inhibiting leukocyte accumulation and expression of TNF-*α*  [92] but also provided protection against ischemia-reperfusion injury in the canine liver [93] and in the rat kidney [94, 95].

After obtaining promising results in animal experiments, rhsTM (ART-123, Recomodulin) proceeded to clinical trials. The efficacy and safety of rhsTM for the treatment of DIC has been investigated in a multicenter, randomized, double-blind phase III clinical trial [9]. This clinical trial was designed to compare rhsTM (0.06 mg kg^−1^ for 30 min once day) with heparin (8 U kg^−1^ h^−1^ for 24 h) in 234 DIC patients. Their underlying diseases consisted of infection and/or hematologic malignancy. During a 6-day course of treatment, half of the patients received rhsTM, while the rest received heparin. The DIC resolution rate at day 7 was significantly better in patients receiving rhsTM (66.1%) than in those receiving heparin (49.9%). The disappearance rate of bleeding symptoms at day 7 was also better in patients receiving rhsTM (35.2%) than in those receiving heparin (20.9%). Remarkably, in DIC resulting from infection, rhsTM treatment improved the mortality rates at day 28 to a greater degree than did heparin treatment (rhsTM 28.0%; heparin 34.6). In the case of DIC resulting from hematologic malignancy, the mortality rates were 17.2% for the rhsTM group and 18.0% for the heparin group. Greater decreases in plasma thrombin-antithrombin complex levels and D-dimer levels were observed in patients treated with rhsTM. Importantly, rhsTM treatment showed a better safety profile with a lower incidence of bleeding-related adverse events. In 2008, rhsTM has been approved in Japan for the treatment of DIC. As of May 2011, 4,260 DIC patients (2,588 infections, 1,121 hematologic malignancies, 93 solid tumors, and 458 miscellaneous underlying diseases) were treated with rhsTM, according to the Asahi Kasei Pharma that has marketed this drug in Japan.

Importantly, a retrospective subanalysis that focused only on the 80 patients having infection as the underlying disease [7] confirmed the usefulness of rhsTM in the treatment of DIC associated with infection (DIC resolution rates (rhsTM: 67.5%; heparin: 55.6%) and 28-day mortality rates (rhsTM: 21.4%; heparin: 31.6%)). In the USA, a phase II clinical trial involving 750 patients has been completed as of May 2011 that compared rhsTM with placebo for the treatment of sepsis with DIC (NCT00487656), thereby awaiting the results publicized. The Asahi Kasei Pharma America reports that they will proceed to phase III clinical trials.

## 7. Concluding Remarks

We are beginning to understand that the crosstalk and interplay that occur between inflammation and coagulation together constitute a critical component driving both the pathogenesis and progression of sepsis. Therapeutic approaches targeting both inflammation and coagulation hold great promise for the treatment of patients suffering septic shock. While APC is the first to be successfully administered under a clinical setting, TM is expected to represent the 2nd generation of effective biologic drugs that target both inflammation and coagulation in septic patients.
